# Shared Ancestry of Symbionts? Sagrinae and Donaciinae (Coleoptera, Chrysomelidae) Harbor Similar Bacteria

**DOI:** 10.3390/insects3020473

**Published:** 2012-05-07

**Authors:** Gregor Kölsch, Dimitra Synefiaridou

**Affiliations:** Zoological Institute, Molecular Evolutionary Biology, University of Hamburg, Martin-Luther-King-Platz 3, 20146 Hamburg, Germany; E-Mail: dimitrasynefiaridou@gmail.com

**Keywords:** Donaciinae, Sagrinae, Enterobacteriaceae, symbiosis, cocoon formation, Anobiidae, Cerambycidae, *Bromius obscurus*, evolution

## Abstract

When symbioses between insects and bacteria are discussed, the origin of a given association is regularly of interest. We examined the evolution of the symbiosis between reed beetles (Coleoptera, Chrysomelidae, Donaciinae) and intracellular symbionts belonging to the Enterobacteriaceae. We analyzed the partial sequence of the 16S rRNA to assess the phylogenetic relationships with bacteria we found in other beetle groups (Cerambycidae, Anobiidae, other Chrysomelidae). We discuss the ecology of each association in the context of the phylogenetic analysis. The bacteria in *Sagra femorata* (Chrysomelidae, Sagrinae) are very closely related to those in the Donaciinae and are located in similar mycetomes. The Sagrinae build a cocoon for pupation like the Donaciinae, in which the bacteria produce the material required for the cocoon. These aspects support the close relationship between Sagrinae and Donaciinae derived in earlier studies and make a common ancestry of the symbioses likely. Using PCR primers specific for fungi, we found *Candida *sp. in the mycetomes of a cerambycid beetle along with the bacteria.

## 1. Introduction

Symbioses are an integral part of life on earth. Associations between two partners with mutual benefit can include plants, animals, fungi or bacteria. Symbioses increase productivity or render the colonization of new habitats possible [1]. Symbioses of insects and bacteria (and to a lesser extent fungi) have attracted considerable interest recently [2,3,4], although many systems have been known for decades [5]. Besides the investigation of functional interactions, the origin of symbioses have long been of interest [5,6,7,8]. Many symbioses are old in evolutionary terms, some are younger [9]. They can sometimes be traced back to the origin of the host group, and can even shed light on phylogenetic relationships [10,11,12,13].

**Figure 1:**
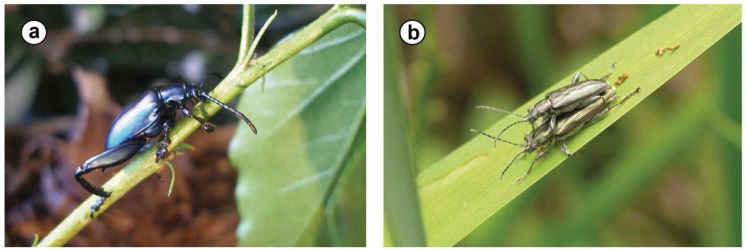
Representatives of (a) the Sagrinae (*Sagra femorata*, 22 mm long) and (b) the Donaciinae (pair of *Donacia* cf. *subtilis* Kunze, 1818, 10 mm long)

Our research focuses on bacteria that belong to a clade of Enterobacteriaceae (gamma-proteobacteria) [14] that may be particularly adapted to form symbioses and may be a group in which such associations diversified [15]. They are associated with reed beetles (Coleoptera, Chrysomelidae, Donaciinae; Figure 1b). Reed beetle larvae pupate under water in the sediment in a self-made cocoon. The material for this cocoon is produced by the bacteria which are located within the cells of four large blind sacs at the larval foregut [16]. The formation of the cocoon is a key adaptation enabling the reed beetles to colonize wetlands permanently [17]. This symbiosis is peculiar with respect to the interaction between the partners, because it is not predominantly a nutritional one as occurs in most known systems, in which the bacteria provide nutrients underrepresented in the diet of the insect [18,19]. This makes an elucidation of the evolutionary origin particularly attractive. It is likely that the association between reed beetles and their symbionts did not establish instantaneously from two completely independent organisms, but rather evolved from an ancestral association. If this is the case, it should be possible to find a similar symbiosis among other Coleoptera.

Therefore, the aim of this study was to search for similar associations and closely related bacteria in other beetle taxa. Our search was guided by three criteria: phylogenetic proximity of the host, similarity and/or location of the morphological structures (mycetomes containing bacteria), and the formation of a cocoon for pupation of the beetle larvae. Four taxa fulfilling at least one criterion were chosen (Table 1). We used approximately 1100 base pairs of the DNA sequence coding for the 16S ribosomal subunit of bacteria in order to assess the phylogenetic relationship between symbionts of these taxa and symbionts of the Donaciinae. For the Anobiidae and the Cerambycidae, an association with yeast-like fungi is known [5]. Therefore, we additionally used specific primers to amplify fungal DNA and thereby to characterize the symbiotic relationships more fully.

**Table 1:** Species used in this study and details with respect to the criteria used for the selection.

Species	Taxonomic Affiliation	Mycetomes	Cocoon Formation?
*Sagra femorata*(Drury 1773)	Chrysomelidae: Sagrinae; closely related to Donaciinae [20,21]	larvae: four blind sacs ‘at the cranial end of the ventriculus’ [22]	yes, for example within the root of the host plant (Kudzu *Pueraria lobata* and others; [23])
*Bromius obscurus*(L. 1758)	Chrysomelidae: Eumolpinae	(a) ‘blind sacs’ differing in morphology between adults and larvae (but containing the same intracellular bacteria) at the beginning of the midgut; (b) short blind ending tubes (‘crypts’) at the midgut, close to the Malpighian tubules, containing bacteria in their lumen [24]	no, larvae are root feeders that pupate in the soil
*Stegobium paniceum*(L. 1758)	Anobiidae	‘evaginations’ at the cranial end of the midgut with intracellular yeast cells, smaller in the imago than in the larva [5]	yes, largely made up of substrate material
Unidentified species	Cerambycidae	mycetomes at the cranial end of the midgut, varying in size between species, and containing yeasts in the cells [5]	no, larvae pupate in the wood they are feeding in

## 2. Results and Discussion

### 2.1. Sequence Statistics

Fungi-specific primers amplified a DNA fragment 1084 base pairs long from the mycetomes of cerambycid beetles (specimen 4), which revealed the presence of fungi related to *Candida* sp. according to a BLAST search in the NCBI GenBank (>95 sequence similarity, 100% in the 18S section of *Candida pseudorhagii*, accession no. EF120587). None of the other samples yielded specific PCR products of fungal DNA.

Bacterial symbionts were found in all the specimens that were analyzed, including the one that was positive for fungi. The nucleotide sequences were unambiguous. The sequences obtained were between 1110 and 1200 base pairs long. The GC-content of two 16S sequences was unusually low (Table 2): 47.9% in *Sagra* symbionts and 44.9% in *Bromius* (blind sacs) symbionts. The nucleotide composition was heterogeneous between sequences (chi-square test; Table 3). This was attributable to the *Bromius* (blind sacs) sequence. Its omission led to homogenous base composition. 

### 2.2. Phylogenetic Analysis

**Figure 2:**
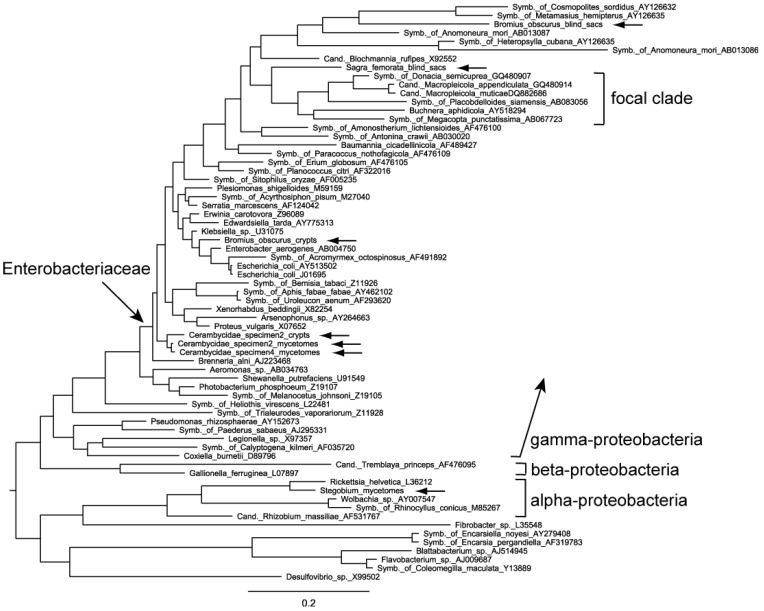
Phylogenetic position of the bacteria analyzed in the present study (horizontal arrows), based on partial 16S rRNA sequences (dataset ‘bacterial phylogeny’). The tree was constructed using the RNA7D model in PHASE and rooted at the base of a clade consisting of the beta- and gamma-proteobacteria. It represents a 50% majority rule tree with branch lengths estimated by the software. GenBank accession numbers are given. ‘Symb._of’ means ‘symbiont of’. The ‘focal clade’ is the branch that contains the symbionts of the Donaciinae. It does not have a formal systematic meaning, and its members are not identical in our two datasets (see text).

**Figure 3:**
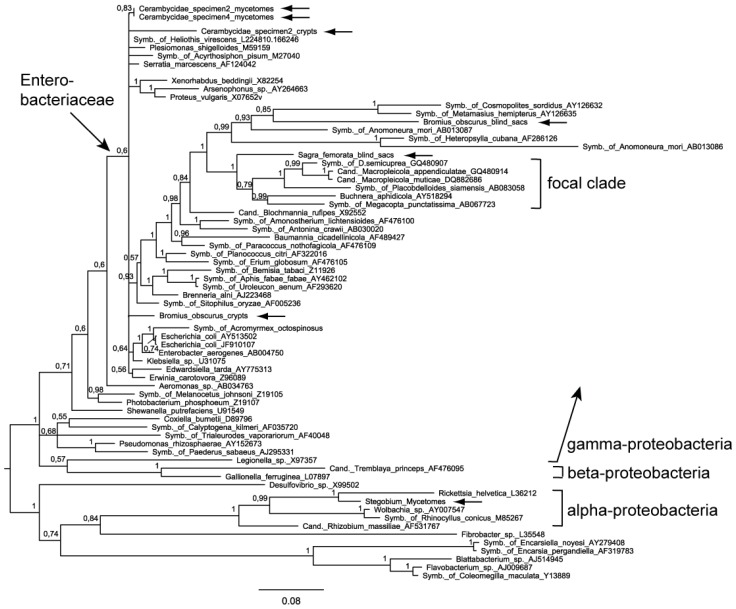
Phylogenetic position of the bacteria analyzed in the present study (horizontal arrows), based on partial 16S rRNA sequences (dataset ‘bacterial phylogeny’). The tree was constructed using MrBayes. Posterior probabilities are given to the upper left of each node. For further explanations, see Figure 2.

In our phylogenetic analysis we concentrated on the trees produced by PHASE (software specifically designed for rRNA data, taking into account secondary structure, Figure 2) and MrBayes (software that in addition allowed us to effectively use the gap information, Figure 3). Apart from minor incongruences between the trees constructed with different phylogenetic methods, the placement of the sequences of interest to us was very similar.

**Table 2:** Origin, GenBank accession numbers and the relative GC-content (16S rRNA only) of the sequences obtained in the present study

Organism	GenBank Accession Number				
	Organ/Tissue	Bacteria (16S)	Fungi (18S/28S)		%GC (16S only)
Cerambycidae specimen 2	mycetomes	JQ805034		JQ805028	53.2%
	crypts	JQ805035			52.1%
Cerambycidae specimen 4	mycetomes	JQ805036			52.3%
*Sagra femorata*	blind sacs	JQ805032			47.9%
*Bromius obscurus*	blind sacs	JQ805030			44.9%
	crypts	JQ805033			53.7%
*Stegobium paniceum*	mycetomes	JQ805029			52.1%

**Table 3:** Chi-squared test for homogeneity of nucleotide frequencies among the sequences obtained in the present study

Sequences Analyzed	Chi-squared Value	df	Probability
(a) including *Bromius* (blind sacs)	33.40	18	p ≤ 0.05
(b) excluding* Bromius* (blind sacs)	11.97	15	not significant

The sequence obtained from *Stegobium* mycetomes differs widely from those of the remaining species. The BLAST search showed that it is closely related to *Rickettsia*.

Bacteria from the mycetomes of Cerambycidae clustered together even though they came from different individuals, if not species. The sequence from the Cerambycidae crypts was most closely related to them. Their exact position within the clade of Enterobacteriaceae is difficult to characterize but they do not belong to a clade which we term ‘focal clade’. We use this term as a concise circumscription of the branch that contains the symbionts of the Donaciinae (see below). It does not have a formal systematic meaning, and its members are not identical in our two datasets.

**Figure 4:**
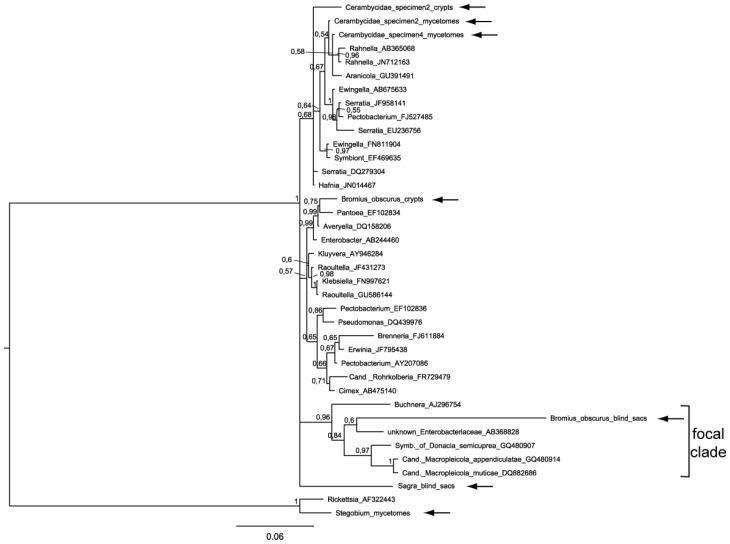
Relationship to closely related taxa of the bacteria analyzed in the present study (horizontal arrows), based on partial 16S rRNA sequences (dataset ‘Enterobacteriaceae’). The tree was constructed using MrBayes. Posterior probabilities are given to the upper left of each node. For further explanations, see Figure 2.

Bacteria from the *Bromius* crypts are positioned among intestinal, extracellular bacteria including *Enterobacter* sp. and *Escherichia coli*. The intestinal blind sacs of *Bromius* contain bacteria that are similar to some other intracellular endosymbionts of insects (Figure 2). In our trees, this taxon is a member of a sister-clade to the group that contains the Donaciinae symbionts. In the phylogenetic tree based on the ‘Enterobacteriacae’ dataset and obtained by using MrBayes (Figure 4), these blind sac symbionts appear to be member of the ‘focal clade’, but with a conspicuously long branch.

Bacteria from the blind sacs of *Sagra* were positioned very closely to the group of Donaciinae symbionts, which are contained in the ‘focal clade’ (see Figure 2 and Figure 3). Other members of this clade as represented in Figure 2 and Figure 3 are three intracellular endosymbionts: *Buchnera aphidicola* Munson *et al*., 1991 is the primary endosymbiont of aphids, transmitted vertically *via* the aphid ovary. The bacterium-aphid symbiosis has a nutritional basis [18]. The symbiont of *Megacopta punctatissima* (Montandon, 1894), the Japanese common plataspid stinkbug, utilizes a unique mechanism of symbiont transmission involving a so-called “symbiont capsule” [25]. For the symbiont of *Placobdelloides siamensis* (Oka, 1917), a hematophagous glossiphoniid leech, the biological function is not known [26], but it consistently appears as one of the best hits in BLAST searches using DNA sequences of symbionts of the Donaciinae. Taken together, the *Sagra* symbionts are not the closest relatives of Donaciinae symbionts according to our dataset of 16S rRNA sequences. In the tree based on the ‘Enterobacteriacae’ dataset, the phylogenetic relationships of the *Sagra* symbiont are not resolved, but an affiliation with the focal clade is not contradicted (Figure 4).

### 2.3 Pattern of Nucleotide Substitutions

The uncertainties with respect to the placement of *Bromius* (blind sacs) and *Sagra* symbionts led us to take a more detailed look at the sequences. There are differences in the mutation pattern in the conserved and variable regions. The *Bromius* symbiont has a lower proportion of base differences in the variable regions compared to the focal clade. For the Enterobacteriaceae dataset this is statistically significant (Fisher’s exact test, p ≤ 0.01; Table 4). For the *Sagra* symbionts there is no such difference, rather a tendency in the opposite direction (Table 5).

### 2.4. Evaluation of the Phylogenetic Trees

The different methods we used to position the taxa we investigated in the bacterial phylogenetic tree yielded very similar results. The incongruences encountered concern the placement of the bacterium found in the intestinal blind sacs of *Bromius* (*Bromius* blind sac symbionts BBSS). It is justified to assume that the tree structure revealed by the analyses in MrBayes and PHASE is most realistic. These two software products can adequately handle the peculiarities of an (our) rRNA data set. They take into account the secondary structure of the RNA molecule, which affects the mutation rate at individual nucleotide positions. Moreover, a detailed inspection of the alignment revealed peculiarities that have to be taken into account. Gaps are an important element that contributes considerably to the dissimilarity between the sequences of BBSS and those of the members of the focal clade. The inclusion of the binary matrix coding the presence and absence of gaps into the MrBayes analyses removed the BBSS from the focal clade. The maximum likelihood analysis in PAUP* does not allow for the consideration of gap information. The maximum parsimony analysis is particularly sensitive to the heterogeneity of the base composition, which inevitably occurs in a data set containing a broad spectrum of bacterial species. Most importantly, the GC-content differs between free-living and intracellular endosymbiotic species (references given in [17]). The GC-content of the DNA sequences we obtained during the present study is largely homogeneous (chi-squared test). The sequences share relatively low GC-values usually found among intracellular endosymbiotic Enterobacteriaceae, which they clustered together with in our phylogenetic tree. Only the BBSS sequence deviates from the pattern, which is demonstrated by a significant chi-squared test after its inclusion into the data set (Table 3). This difference in base composition might specifically affect the positioning of BBSS in the tree.

There are indications for long branch attraction of BBSS as well. If we use our second set of 16S rRNA sequences (‘Enterobacteriaceae’; Figure 4), which contains only sequences very similar to the ones we obtained, the BBSS appears in the focal clade again, with a strikingly long branch. This can be attributed to the lack of suitable outgroup taxa, which are present in the more comprehensive data set (‘bacterial phylogeny’). One last aspect that can explain the occasional placement of BBSS in the focal clade is the pattern of mutations in the BBSS. Defining conserved and variable regions in the alignment of all 16S rRNA sequences, we find that the BBSS shows an above-average rate of mutations in conserved regions (Table 4). This pattern can be interpreted as an indication of larger evolutionary distance. Mutations in those regions are relatively rare by definition, but here they are overrepresented. In the variable regions, saturation with multiple substitutions at some positions may have led to disproportional similarity to in fact distant taxa, which is the basis of the long branch attraction suspected here.

That means that the BBSS has to be placed outside the group most closely related to the focal clade containing Donaciinae symbionts. The *Sagra* symbiont, on the other hand, is firmly allied with that clade, as demonstrated by the high posterior probability of the branch (*Sagra* + focal clade) (Figure 3). If *Sagra* is a very close relative of the Donaciinae, then the placement of *Buchnera* and two other symbionts of invertebrates between their symbionts is irritating. It can be due to true common ancestry, convergent sequence evolution or horizontal gene transfer. Compared to the three Donaciinae symbionts, *Buchnera* has fewer nucleotide substitutions than the *Sagra* symbionts, but this difference occurs only in variable regions (123 substitutions in *Buchnera vs*. 164 in *Sagra*, 1 substitution in conserved regions in both cases). One has to keep in mind that the independent evolution of Sagrinae and Donaciinae is an estimated 75–100 million years (origin of the Donaciinae; [27]), and that mutation rates may have differed between lineages.

**Table 4:** Number of nucleotide substitutions in conserved *vs.* variable regions in sequences of *Bromius* (blind sacs) symbionts and members of the focal clade, and the ratio of the two values (variable/conserved). Numbers given are the mean number of substitutions found in pairwise comparisons. The datasets are explained in section 3.3. The ‘focal clade’ is a group of six sequences containing the symbionts of Donaciinae and a few others (see Figure 2 andFigure 3), in the ‘Enterobacteriaceae’-dataset, the focal clade is more restricted (see Figure 4).

Dataset	Taxa analyzed	Conserved regions	Variable regions	Ratio var./cons.
‘Enterobacteriaceae’	Average within focal clade excl. *Bromius*	3	78	29.4
	*Bromius* compared to members of the focal clade	21	116	5.5
‘Bacterial phylogeny’	Average within focal clade	2	154	97.0
	*Bromius* compared to members of the focal clade	5	147	29.5

**Table 5:** Number of nucleotide substitutions in conserved vs. variable regions in sequences of *Sagra* symbionts and members of the focal clade, and the ratio (variable/conserved) (further explanations: see Table 4).

Dataset	Taxa analyzed	Conserved regions	Variable regions	Ratio var./cons.
‘Enterobacteriaceae’	Average within focal clade	9	81	9.2
	*Sagra* compared to members of the focal clade	6	110	19.4
‘Bacterial phylogeny’	Average within focal clade	3	190	64.3
	*Sagra* compared to members of the focal clade	‘1’ (0.3)	122	367.0

### 2.5. Details of the Symbioses and the Biology of the Species Involved

**Figure 5:**
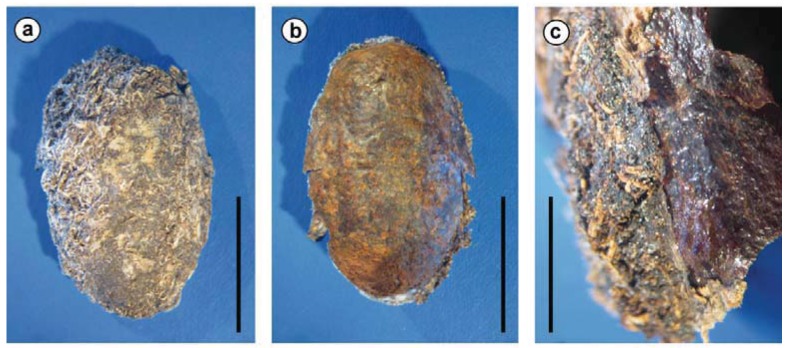
(a) Cocoon of *Sagra femorata* seen from the outside. (b) Inner surface of the cocoon. (c) Wall of the cocoon, revealing its two-layered nature. Under an outer layer, to which substrate particles adhere (left, partly delaminating), there is an irregular, but smooth (shining) inner layer made up of amber-colored material (right). Scale bars: (a), (b) 1 cm, (c) 4 mm.

Sagrinae and Donaciinae are closely related subfamilies within the leaf beetles [20,28]. The genetic similarity of their symbionts further supports their close relationship. These are more closely related to each other than to any other beetle symbiont analyzed in the present study (Figure 2 and Figure 3). In addition to similarity based on DNA sequences, members of both groups build a cocoon for pupation. For the Sagrinae, this is described as a lining of the pupal cell [29] located inside a plant stem/root, but effectively the result is a cocoon that can be removed intact from the substrate (Figure 5). The same holds true for cocoons of Donaciinae, which the larva builds in the sediment attached to the root of the host plant [30,31]. This cocoon formation is peculiar and occurs in no other group of leaf beetles. Stammer [24] described how the bacterial endosymbionts of the Donaciinae produce a secretion the larvae use for building the cocoon. It remains to be investigated if a similar function can be attributed to the Sagrinae symbionts. It should be emphasized that the amber-colored material in both groups appears similar. It is a thin, parchment like layer, flexible but brittle when dry. It is clearly more substantial than a surface secretion that can slightly stabilize the pupal chamber, as found in many leaf beetles [32] (and which is present in the initial phase of cocoon formation in the Donaciinae, [31]).

The Sagrinae and Donaciinae also share a common location of the organs that contain the symbionts. There are four blind sacs at the anterior end of the midgut (described as ‘diverticula on the anterior portion of the ventriculus’ in *Sagra* [22]). Admittedly, this is a region where mycetomes can be found in several other insects (see also below). However, the ones found in larvae of Donaciinae and Sagrinae are sac-shaped and have a regular, smooth wall, while usually we find structures resembling acinous glands (e.g., [5,24,33,34]). Taken together, we find evidence for three criteria of homology, namely anatomical position, gross and microscopic morphology and possibly function (if the symbionts are involved in cocoon formation in both groups).

Another chrysomelid beetle included in our study is the species *Bromius obscurus* (Eumolpinae). We used two different sources of bacterial symbionts from adult specimens of that species (after [5,24]). The first one was a ring of short finger-shaped evaginations at the transition of foregut and midgut, for which we used the term blind sacs. The thin tubes contain the same intracellular bacteria as four blind sacs at the same location in the larvae [24]. The bacteria are located in ‘vacuoles’. The second symbiont source was a group of even shorter tubules (‘crypts’) at the end of the midgut that contain in their lumen bacteria of a different shape than those in the blind sacs according to [24]. During the dissection, we took care only to sample the appendices of the gut, not the gut tissue itself or gut content. The clear, unambiguous signal we obtained for our DNA-sequences indicates that we succeeded in this effort to obtain ‘pure’ symbiotic bacteria. The bacteria contained in the two organs are not directly related to the Donaciinae symbionts, in spite of the superficial resemblance of the four *larval* blind sacs. They even belong to two different clades of the Enterobacteriaceae, each clustering with bacteria of similar life style. The ones from the blind sacs are positioned among other intracellular endosymbionts, while the ones from the crypts are related to extracellular intestinal bacteria.

Anobiidae are well known due to their occurrence in stored products (e. g., *Stegobium paniceum* L. and *Lasioderma serricorne* (Fabricius, 1792)). The larvae harbor endosymbiotic yeasts at the anterior end of the midgut in mycetomes [35], which have an irregular shape like an acinous gland. This symbiosis is among the longest known insect symbioses [36]. The only bacterium we were able to detect in this tissue identified as *Rickettsia* sp. by its high sequence similarity to that genus. A determination to species level is not reliable based on 16S rRNA [37]. *Rickettsia* is a widespread symbiont of insects and other metazoans, and the discussion of its role in the symbioses and the evolutionary biological consequences goes beyond the scope of this study. We were unable to detect yeast in the mycetomes. Either the primers did not amplify this specific yeast fungal DNA, or the larvae were devoid of yeast symbionts. The latter is possible, because the laboratory culture had been maintained on nutrient rich artificial food (koi carp pellets) for many generations, making the supplementation by symbionts obsolete. It is known that experimentally sterilized larvae can grow normally if the food contains the vitamins required [38]. Cocoon formation in the Anobiidae is different from that in the Donaciinae. The Anobiidae pupate in a cocoon that is largely made up of fragments of the surrounding substrate and maybe faeces ([39,40,41], unpubl. data for *Stegobium*, Kölsch). Even if some saliva is involved in gluing the material together, the amount of secretion and its final structure are different from that characterizing the cocoon found in Donaciinae and Sagrinae.

Like the symbiosis of the Anobiidae, the associations found in Cerambycidae have long attracted considerable interest from biologists [33,42]. The fungus-specific primers we used amplified DNA from Cerambycidae. The yeast sequence identified in a BLAST search was a *Candida* species, in accordance with the classification of many symbionts of long-horned beetles [7,34,43]. The yeasts provide vitamins to the host rather than digest cellulose [44], as had been suggested earlier (see discussion in [45]). In contrast to many earlier studies that mentioned exclusively yeasts, but in accordance with the study by Grünwald *et al*. [34], we also found bacterial symbionts in cerambycid guts. We specifically sampled the mycetomes at the proximal end and the spherical, nodule-like protrusions (crypts) at the distal end of the larval midgut. The resulting DNA-sequences were clearly legible, which hints at homogeneous populations of bacteria in both structures. It should be emphasized that both fungi and bacteria were isolated from the mycetomes in one specimen, although we are not able to evaluate this coexistence without more detailed data on their abundance and location (intra-/extracellular). The bacteria from crypts and mycetomes were very similar. Moreover, the sequences obtained from the mycetomes of two different individuals were almost identical. This indicates they are a relatively constant element of the gut flora of at least some Cerambycidae. Grünwald *et al*. [34] discovered intracellular bacteria in the gut wall (but not in the mycetomes) of *Tetropium castaneum* (Linnaeus 1758), which are related to *Sodali*s, the endosymbionts of *Glossina* species. This shows that such relatively stable and intimate associations occur in Cerambycidae, although the 16S signature of the symbionts we discovered rules out an affiliation with *Sodalis*. The GC-content in the range of 52–53% indicates that we deal with extracellular, not intracellular endosymbionts (for references on GC-content, see [17]). The entire intestinal bacterial community of insects is usually much more diverse, with most species being located in the gut lumen [46,47,48,49,50,51,52,53,54,55]. However, sometimes few dominant bacterial species are found, like in the cerambycid beetle *Tetropium castaneum* [34].

## 3. Experimental Section

### 3.1. Sample Collection

Larvae of Cerambycidae were collected in a conifer forest, 30 km north of Hamburg, Germany (53.76N, 9.97E). Specimen no. 2 was found under the bark of a fir tree logged about four years before our collection, specimen 4 was recovered from a pine log approximately two years old. Specimens of *Sagra femorata* from Thailand, still in their cocoon, were bought from a commercial insect farm. One larva among them was used for the present study. *Stegobium paniceum* was obtained from a laboratory culture of beetles that originally had been collected in southern Germany, kindly provided by J. Steidle, University of Stuttgart-Hohenheim, Germany. They were reared on koi carp food pellets at 26° C. *Bromius obscurus* was collected from the food plant *Epilobium hirsutum* (Linnaeus 1758) 20 km east of Hamburg, Germany (53.52N, 10.31E). *Donacia semicuprea* was collected from *Glyceria maxima* Hartmann, 1919 at a pond in Hamburg, Germany (53.63N, 9.90E) (GenBank accession number JQ805031). For the other two symbionts of Donaciinae (*Candidatus* Macropleicola appendiculatae and *Candidatus* Macropleicola muticae), the sequences from [14] and [17] were used.

### 3.2. Laboratory Procedures

The following insect tissues were removed for bacterial analysis. For Cerambycidae, two different larval structures were used: mycetomes at the proximal end of the midgut and nodule-like protrusions at the distal end of the midgut, which will be referred to as crypts (see [34] for a photograph of the intestinal tract of a larva). In order to obtain enough material, 20–30 crypts were pooled from each individual. For *Sagra*, four blind sacs found at the larval foregut [22] were used. A ring of mycetomes [5,56] was dissected from the proximal end of the larval midgut of *Stegobium*. *Bromius* samples included the ring of numerous finger-shaped protrusions at the proximal end of the midgut ([24]), referred to as blind sacs, and a group of shorter tubules at the end of the midgut, referred to as crypts. Malpighian tubules of *Donacia semicuprea* were sampled as described in [17]. Tissues were either stored frozen (Cerambycidae, *Bromius*) or animals were freshly killed just prior to use (*Sagra*, *Stegobium*). Dissections were carried out under ethanol. The DNA was extracted by using the Qiagen DNeasy kit with the general protocol for animal tissue (small elution volume: 25–50 μL). The DNA coding for 16S ribosomal RNA was partially amplified in a touchdown polymerase chain reaction (PCR) with initial denaturation at 95 °C for 10 min and, in each cycle, denaturation at 95 °C for 30 s, annealing for 1 min and extension at 72 °C for 1 min. Annealing commenced at 60 °C, decreased by 0.5 °C per cycle for 14 cycles and then remained at 53 °C for another 21 cycles, followed by a final extension step at 72 °C for 7 min. The primers used were bac357for (5'-CTCCTACGGGAGGCAGCAG-3') and bac1492rev (5'-TACGGYTACCTTGTTACGACTT-3') [17]. The primers 18S.1 (5'-TCGTAGTCTTAACCATAAACTA-3’; courtesy of S. Dobler, Hamburg, and S. Kelley, San Diego) and ITS4r (5'-TCCTCCGCTTATTGATATGC-3'; [57]) were used to amplify a section of fungal DNA spanning from the 18S rRNA gene to the 28S rRNA gene. This primer combination proved to be universal for fungi without amplifying DNA from other taxa (unpubl. data; Dobler and Kelley). The PCR conditions were: initial denaturation at 95 °C for 2 min and, in each of 35 cycles, denaturation at 95 °C for 45 s, annealing at 47 °C for 1 min and extension at 72 °C for 1 min. This was followed by a final extension step at 72 °C for 5 min. Sequencing in both directions was outsourced to a commercial provider (GATC Biotech, Constance, Germany). 

### 3.3. Phylogenetic Analysis

Two datasets were created for the construction of phylogenetic trees. The first one (‘Enterobacteriacae’) contained our sequences and between five to ten sequences most similar to each of them according to a BLAST search in GenBank (representatives of all taxa found among the first 100 hits). For the second one (‘bacterial phylogeny’) we retrieved sequences from GenBank spanning a wide range of bacterial systematics. Taxon selection was almost identical to the one in [14] to facilitate comparisons. Emphasis was laid on bacteria living as endosymbionts in invertebrates, especially insects, but several free-living species were included as well. Accession numbers for sequences retrieved from GenBank are given in Figure 2 through Figure 4. The fungus found in the cerambycid beetle was also identified using a BLAST search in the GenBank nucleotide database. The sequences were viewed and manipulated using BioEdit version 7.0.9.0 [58].

In an initial conventional approach, sequences were aligned by using MUSCLE version 3.6 [59] with default settings. The resulting alignment required moderate additional editing. In a second approach, the software RNAsalsa 0.8.1 [60] was used to simultaneously derive a secondary structure of the RNA molecule and to obtain a structure-guided alignment. For this purpose, the secondary structure of the 16S rRNA of *Escherichia coli* (accession number J01695; [61]) was used as a reference constraining the analysis in RNAsalsa. Both datasets were truncated to obtain sequences of the same length.

The structural model and the alignments from both MUSCLE and RNAsalsa were used for phylogenetic analyses in PHASE 2.0 (http://www.bioinf.manchester.ac.uk/resources/phase/). Two different models of sequence evolution were used for unpaired and paired nucleotides (RNA6B and RNA7D, respectively; for details of the models, see the software manual available online). A discrete gamma distribution of substitution rates with six gamma categories and without invariant sites was assumed. For the Bayesian inference of the phylogeny, the parameters for the MCMC runs were chosen according to recommendations in the software manual. The burnin was set to 150,000 iterations, compared with 600,000 sampling iterations with a sampling period of 100. The relevant parameters rapidly converged, and independent runs yielded the same results. By using the mcmcsummarize routine of the PHASE package, a consensus tree showing all clusters with a Bayesian posterior probability of 50% or higher was calculated.

The phylogenetic analysis was also carried out using PAUP* 4.0b10 [62] (maximum parsimony MP, maximum likelihood ML) with settings as follows (default settings were used for all options not mentioned). MP: heuristic search using the TBR algorithm, random addition of sequences with 1,000 replicates, gaps treated as fifth character state, number of trees in memory (‘maxtrees’) automatically increased; bootstrap analyses using fast stepwise addition with 1,000 replicates. ML: substitution model GTR+I+G (selected in Modeltest 3.7, [63]), with base frequencies, shape parameter and proportion of invariable sites user defined (Modeltest); heuristic search using the TBR algorithm, random addition of taxa with ten replicates.

Additionally, the phylogeny was analyzed by using MrBayes 3.1.2 [64] with 3,000,000 generations, sample frequency = 100 and burnin set to 7500 samples; the GTR model of nucleotide substitution was used with a partition into loop and stem regions with application of a 4/4 and a doublet model, respectively, and priors unlinked between partitions. Five different temperatures were tested (0.05, 0.1, 0.2 = default, 0.3, 0.5) and at least three independent runs were performed for each combination of parameters. In order to use the gap information in MrBayes, we created a binary data matrix by replacing alignment gaps by “1” and every nucleotide by “0”. The final dataset contained both the sequence information and the binary gap information.

### 3.4. Variable and Conserved Regions, Nucleotide Frequencies

The number of nucleotide substitutions between the sequences was calculated. We defined variable and conserved regions in the ‘bacterial phylogeny’ data set using BioEdit (changed settings: maximum segment length: 8, maximum average entropy: 0.25, maximum entropy per position: 0.25). The number of differences between the sequences in variable and conserved regions, respectively, was determined by using the software MEGA version 5 [65]. From these matrices, the mean number of differences was calculated between a certain sequence of interest and the sequences in a cluster it was compared to. The homogeneity of the nucleotide frequencies between the sequences produced in the present study was tested by using the chi-squared test implemented in DAMBE 4.0.36 [66].

## 4. Conclusions

The aim of this study was to elucidate the evolutionary origin of the symbiosis of intracellular bacteria and the reed beetles. Since it is not possible to achieve a comprehensive analysis of all symbioses involving beetles, we concentrated on a few systems selected according to criteria outlined in the introduction. Among them, the Sagrinae harbor the bacteria most closely related to the symbionts of Donaciinae. Such a close relationship is further supported by the similarity in the morphology and position of the mycetomes as well as a potential role of the bacteria in the formation of the cocoon by the beetle larvae. This cocoon formation *per se* to the best of our knowledge is a synapomorphy of Sagrinae and Donaciinae. Among the bacterial taxa selected for our phylogenetic analysis, three other invertebrate symbionts are classified as even more closely related to the symbionts of Donaciinae. This can be due to true common ancestry, convergent sequence evolution or horizontal gene transfer. If the 16S rRNA we chose to investigate cannot fully and in a reliable manner resolve the relationship of these closely related taxa, a more comprehensive approach involving more genes like multi-locus sequence analysis [67] may help to achieve this. The fundamental question underlying this study was that of the origin of the symbionts of the Donaciinae. Assuming a shared ancestry with the symbionts of Sagrinae, this problem is partly solved. However, at the same time it is shifted to a hypothetical common ancestor of both bacterial lineages, which may have established in an early lineage of the Chrysomelidae. It should be noted that in other closely related subfamilies (Criocerinae, Bruchidae/Bruchinae) there is no indication of similar symbioses ([68,69], cited in [70]).
